# Impact of Low Hepatitis Delta Virus Screening Rates on the Accurate Estimation of Seroprevalence in Florida: A Retrospective Observational Study

**DOI:** 10.7759/cureus.79307

**Published:** 2025-02-19

**Authors:** Sean-Patrick A Prince, Sirisha Gaddipati, Paul Martin, Yalda Zarnegarnia, Patricia Denise D Jones

**Affiliations:** 1 Internal Medicine, University of Miami Miller School of Medicine at Holy Cross Hospital, Miami, USA; 2 Internal Medicine, Jackson Health System, Miami, USA; 3 Internal Medicine and Hepatology, University of Miami Miller School of Medicine, Miami, USA; 4 Public Health Sciences, University of Miami Miller School of Medicine, Miami, USA; 5 Sylvester Comprehensive Cancer Center, University of Miami Miller School of Medicine, Miami, USA

**Keywords:** hbv, hdv, hepatitis b virus, hepatitis delta, screening

## Abstract

Background: Hepatitis delta virus (HDV) is a hepatotropic virus that can accelerate the progression of liver disease to cirrhosis and is associated with an increased risk of liver-related mortality and liver cancer. As a defective single-stranded RNA virus, it can only occur with acute hepatitis B or in patients with chronic hepatitis B virus (HBV) infections. In light of these risks, current screening guidelines recommend universal screening in all hepatitis B surface antigen (HBsAg)-positive individuals and those at risk for HDV. Unfortunately, HDV seroprevalence data remains limited due to low rates of HDV screening in the US and worldwide.

Aim: The aim of this study is to determine HDV regional screening rates, prevalence, and associated factors using OneFlorida research data.

Methods: Adult patients aged 18-99 with a previous HBV diagnosis were identified using OneFlorida+ Clinical Research Network data. Using International Classification of Diseases codes, HBV-positive patients were categorized as definite, probable, or possible. This data was then analyzed using a two-sample t-test to determine possible associations with race, gender, or age.

Results: We found that among patients who were tested, HDV seroprevalence was 4.7%. Of the total of 8,744 individuals included in the study, only 225 (2.6%) underwent HDV antibody testing. Although there was no significant difference in age or race, males were more likely to undergo HDV testing.

Conclusion: Despite a low screening rate, HDV seroprevalence in Florida was approximate to previous estimations of HDV positivity.

## Introduction

Hepatitis delta virus (HDV) causes the most severe liver disease among the hepatotropic viruses. First detected in 1977 [1], HDV is a “defective” single-stranded RNA virus that relies on the hepatitis B surface antigen (HBsAg) for entry into hepatocytes, as well as for its assembly and propagation. Although HDV infection can occur simultaneously with acute hepatitis B virus (HBV) infection, this scenario typically leads to spontaneous seroconversion of both viruses in adults [2]. Chronic HDV typically results from acute HDV infection in patients with chronic HBV. Co-infection with HDV accelerates progression to cirrhosis and is associated with a significantly increased risk of cirrhosis, liver decompensation, liver-related mortality, liver transplant, and hepatocellular carcinoma [3,4].

The European Association for the Study of the Liver (EASL) recommends screening all HBsAg-positive individuals for HDV [5]. The American Association for the Study of Liver Diseases (AASLD) recommends screening those at risk for HDV [6], based on birthplace in regions that are highly endemic for HDV or behavioral risk factors that may increase the risk for HDV such as injection drug use. The global prevalence of HDV is poorly understood because screening rates are generally low; however, the data available demonstrates a high disease burden in Asia and West Africa [7]. Reported HDV prevalence in the US varies widely from 2.2% to 42% [8,9].

Despite societal guidelines recommending screening, reported HDV screening rates in the US are low, ranging from 6.7 to 19.7% [10]. Florida has the fourth highest prevalence of HBV/HDV co-infection in the US, 5.8%, trailing Illinois, 38.1%, New York, 16.3%, and California, 10.5% [8]. In this study, we aimed to characterize regional screening rates and HDV prevalence using OneFlorida, a large multi-centered clinical research consortium.

## Materials and methods

The OneFlorida+ Clinical Research Network, a partnership of 14 health systems that care for 15 million persons, covers over 74% of Floridians. The OneFlorida Data Trust was previously used to examine screening rates for chronic hepatitis C [11-13]. The International Classification of Diseases (ICD)-10 codes (B18.0 and B18.1) or ICD-9 codes (070.22, 070.23, 070.32, or 070.33) were used to identify individuals with HBV. Additionally, the Logical Observation Identifiers Names and Codes (LOINC) codes were used to identify individuals with laboratory data confirming that they were seropositive for HBsAg or had detectable serum HBV DNA. All patients with an ICD-9/10 code or LOINC code were included and categorized as definite, probable, or possible.

Individuals with definite HBV had ICD codes on at least two occasions greater than six months apart and reactive HBsAg or detectable HBV DNA. Those with probable HBV had one ICD code and reactive HBsAg or detectable HBV DNA. All others were categorized as possible HBV. We hypothesized that individuals with multiple visits and more confirmatory laboratory data (definite HBV) might be more likely to be engaged in care than those with fewer visits (possible HBV), which might translate to higher rates of HDV screening. All of these patients had HBV; however, these categories were created to distinguish between individuals with multiple data points supporting their diagnosis of HBV and others with fewer data points and could approximate engagement with the health system as more encounters and laboratory tests suggest increased engagement.

For statistical analysis, R software (version 4.3.1; The R Foundation for Statistical Computing, Vienna, Austria) was used. Continuous variables are reported as median with interquartile range and counts (percentages) are used for categorical demographics. LOINC codes, 30 in total, corresponding to tests for HDV antibody, antigen, genotype, or RNA were used to identify testing for HDV (Supplemental Material 1). Also included were the number of subjects with available HDV results and the percentage of positive and negative cases. To evaluate for an association between age, sex, race/ethnicity, and HDV test order status, we used Chi-squared tests or two sample t-tests. The normality assumption of continuous data was checked and we generated a map, using the package “choroplethrZip”, to show the distribution of HBV patients throughout Florida. The study was approved by the University of Miami Miller School of Medicine and the OneFlorida Institutional Review Boards (approval number #20211222).

## Results

In total, 443 individuals without laboratory data were excluded and we included 8,744 unique individuals, from 425 unique facilities. The median age was 51 years and 57.8% were male. The sample consisted of 43% White individuals, 30.2% Black individuals, 12% individuals of other racial/ethnic backgrounds, 11.4% Asian individuals, and 11.2% Hispanic individuals. According to pre-specified definitions, 1,387 individuals had definite, 1,064 had probable, and 6,293 had possible chronic HBV (see Table 1 for detailed baseline characteristics).

**Table 1:** Baseline Characteristics of Chronic HBV Patients in the OneFlorida+ Clinical Research Network (January 1, 2012-September 30, 2021), Overall and Stratified by Definite, Probable, and Possible Categories (a) a: For HBV diagnosis, we used the International Classification of Diseases (ICD) and Logical Observation Identifiers Names and Codes (LOINC) codes to identify individuals seropositive for hepatitis B surface antigen (HBsAg) or with detectable serum HBV DNA. Individuals with definite HBV had ICD codes on at least two occasions greater than six months apart and reactive HBsAg or detectable HBV DNA. Those with probable HBV had one ICD code and reactive HBsAg or detectable HBV DNA. All others were categorized as possible HBV. b: Individuals with age >89 (n = 23) had age masked to 200. c: LOINC codes corresponding to tests for “Hepatitis delta antibody” (3), “Hep D Ab” (121), “Hepatitis D Virus (HDV) IgM Antibody EIA” (2), “Hepatitis D virus total antibodies, serum” (3), and “Hepatitis D antibody, total” (96) were found within the database. The number of individuals who had each respective test ordered is indicated by the number in parentheses found after the test name. d: Percentage calculated using the number of individuals with tests ordered as the denominator. AI/AN: American Indian/Alaskan Native; HBV: hepatitis B virus; HDV: hepatitis delta virus; IQR: interquartile range; HMO: Health Maintenance Organization

	Overall (N=8744)	Definite Chronic HBV (N=1387)	Probable Chronic HBV (N=1064)	Possible Chronic HBV (N=6293)
Median Age (IQR)^b^	51 (39, 61)	49 (38, 58)	47.5 (36, 58)	52 (40, 62)
Race, n (%)				
AI/AN	18 (0.2)	3 (0.2)	2 (0.2)	13 (0.2)
Asian/Pacific Islander Individuals	1013 (11.6)	199 (14.4)	151 (14.2)	663 (10.5)
Black Individuals	2645 (30.2)	448 (32.3)	355 (33.4)	1842 (29.3)
White Individuals	3758 (43)	494 (35.6)	393 (36.9)	2871 (45.6)
Multiple Races	108 (1.2)	16 (1.2)	17 (1.6)	75 (1.2)
Other Individuals	1048 (12)	219 (15.8)	130 (12.2)	699 (11.1)
Unknown/Refused	154 (1.8)	8 (0.6)	16 (1.5)	130 (2.1)
Payor, n (%)				
None/Self-pay/Charity	709 (12.5)	56 (5.9)	102 (13.3)	551 (13.9)
Medicaid	966 (17)	140 (14.8)	139 (18.1)	687 (17.3)
Medicare	1101 (19.3)	157 (16.6)	83 (10.8)	861 (21.6)
Private Insurance	2083 (36.6)	525 (55.4)	331 (43.1)	1227 (30.8)
HMO	435 (7.6)	32 (3.4)	61 (7.9)	342 (8.6)
Tricare	5 (0.1)	0	0	5 (0.1)
Corrections	68 (1.2)	10 (1.1)	9 (1.2)	49 (1.2)
Other	327 (5.7)	28 (3)	43 (5.6)	256 (6.4)
Hispanic Ethnicity, n (%)	981 (11.2)	114 (8.2)	116 (10.9)	751 (11.9)
Sex, n (%)				
Female	3694 (42.2)	574 (41.4)	451 (42.4)	2669 (42.4)
Male	5050 (57.8)	813 (58.6)	613 (57.6)	3624 (57.6)
HDV Tests Ordered, n (%)^c^	225 (2.6)	14 (1)	21 (2)	190 (3)
HDV Tests Results, n (%)^d^				
Positive	6 (2.7)	2 (14.3)	1 (4.8)	3 (1.6)
Negative	121 (53.8)	9 (64.3)	20 (95.2)	92 (48.4)
Not Resulted	98 (43.6)	3 (21.4)	0	95 (50)

**Figure 1:**
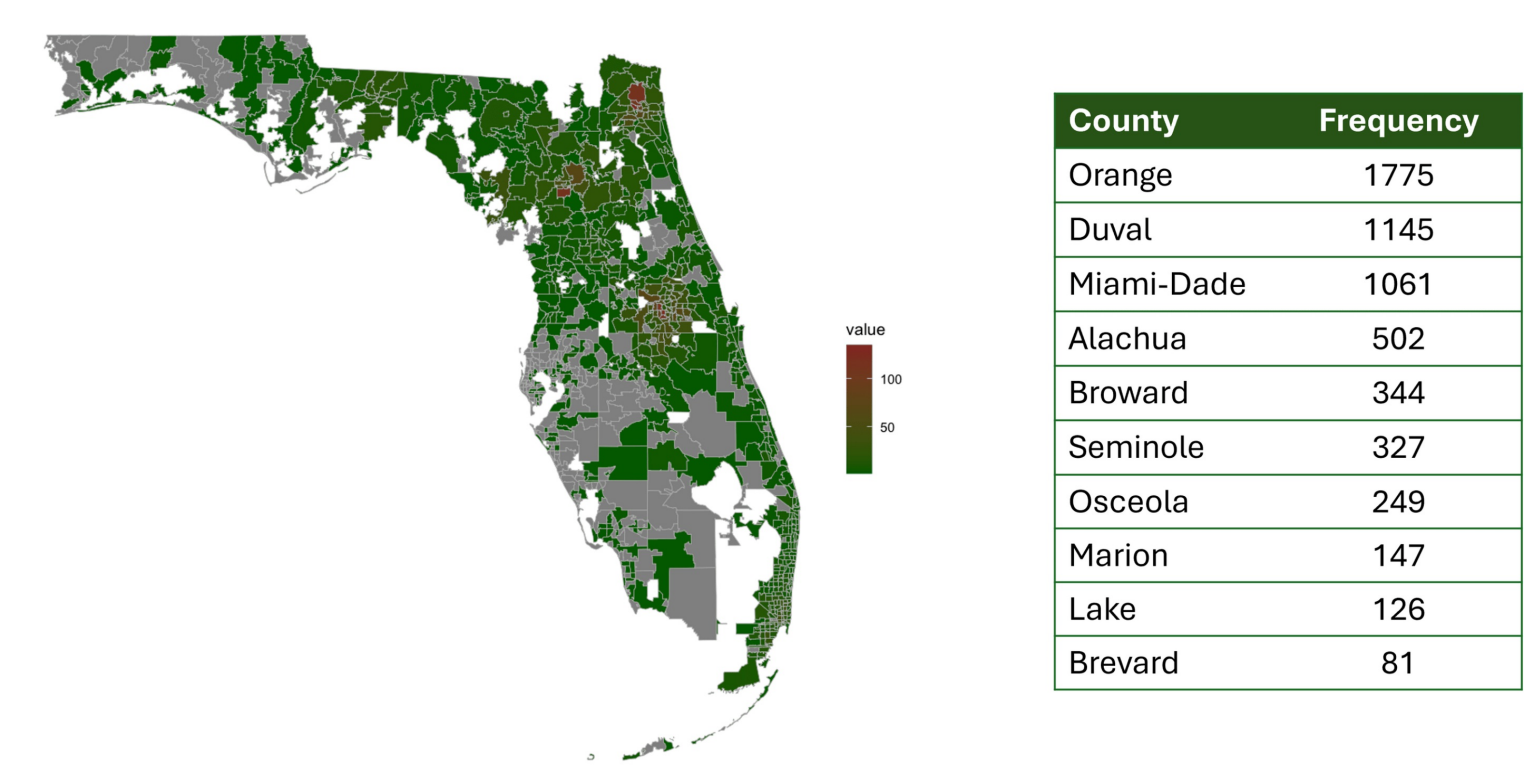
Statewide Distribution of Hepatitis B Cases in Florida This map illustrates the distribution of chronic hepatitis B (HBV) cases across Florida. Areas in white represent areas without mappable zip codes, e.g., the Everglades. Areas in grey represent zip codes not included in the OneFlorida+ database. Image Credits: Yalda Zaldegarnia, the author.

The primary payor was private insurance in 36.6% of the sample, followed by Medicare in 19.3%, Medicaid in 17%, and Health Maintenance Organization (HMO) in 7.6%. There was no reported insurance for 12.5% of the sample while 1.2% had corrections reported as the primary payor. Patients came from 736 mappable zip codes distributed throughout the state, with the highest HBV burden in Orange (n = 1,775), Duval (n = 1,145), and Miami-Dade (n = 1,061) counties (see Figure 1).

LOINC codes, "22330-5", "13248-0", "40727-0" and "44938-9", which correspond to HDV antibodies, were found in the dataset. LOINC codes for other HDV tests, e.g. HDV RNA, antigen, and genotype, were not found in the dataset. HDV antibody was ordered in 225 individuals, 2.6% of the sample, with a median age of 49 years. When stratified, HDV testing was ordered in 1,387 (1%) of those with definite, 1,064 (2%) of those with probable, and 6,293 (3%) of those with possible chronic HBV. Of those 225 persons for whom HDV tests were ordered, 96 were White individuals (42.7%), 55 were Black individuals (24.4%), 37% were Others (16.4%), and 29 were Asians (12.9%); 31 were Hispanic individuals (13.8%) and 148 were male (65.8%). In 98 individuals with HDV test orders, results were unavailable. Results for HDV were available for 127 individuals and six individuals had positive HDV tests, reflecting HDV positivity of at least 4.7% among those tested.

There was no significant difference in median age between those with HDV orders, median age 49 years (range 19-87), and those without orders, median age 51 years (range 18-89). A two-sample t-test was conducted to compare the ages of individuals with and without lab orders, yielding a p-value of 0.71. The normality assumption was assessed using visual inspection methods and the Shapiro-Wilk test, which yielded a p-value of 0.21. Of those with HDV labs, 148 were male (65.8%) whereas 4,902 males (57.5%) made up the sample of those without HDV labs. There was a significant association between order status and gender (p = 0.02). There was no significant association between race and HDV order status (p = 0.15). However, when stratified by sex, a significant association was found between race (Asian, Black, Other, and White) and HDV order status in males using the Chi-squared test (p = 0.02), but not in females (p = 0.19) (see Table 2).

**Table 2:** Proportion of HDV Tests Ordered by Race, Overall and Stratified by Gender Values are presented as n (%), where n represents the number of individuals, and (%) indicates the proportion within each racial/ethnic category. HDV: Hepatitis delta virus

Group	Asian Individuals	Black Individuals	White Individuals	Other Individuals	p-value
Males and Females Combined					0.15
HDV not ordered	967 (97.1%)	2,590 (97.9%)	3,662 (97.4%)	1,152 (96.7%)	
HDV ordered	29 (2.9%)	55 (2.1%)	96 (2.6%)	39 (3.3%)	
Males					0.02
HDV not ordered	403 (96.2%)	1483 (97.4%)	2330 (97.4%)	601 (95.4%)	
HDV ordered	16 (3.8%)	39 (2.6%)	60 (2.6%)	29 (4.6%)	
Females					0.19
HDV not ordered	564 (97.7%)	1107 (98.6%)	1332 (97.4%)	551 (98.2%)	
HDV ordered	13 (2.3%)	16 (1.4%)	36 (2.6%)	10 (1.8%)	

## Discussion

Our study represents the first attempt to exclusively determine HDV seroprevalence in Florida, a state with a significant HBV burden. Despite our HDV positivity rate of 4.7% exceeding previous estimates ranging from 2.2% to 3.4% reported from analyses of commercial laboratory and Veterans Affairs data, respectively [10,14], it is consistent with newer research from one systematic review and an analysis of claims data noting national HDV positivity rates of 4.5% [3] and 4.6% [8], respectively. These studies also correspond with a recent meta-analysis of 1.971 million persons estimated to have chronic HBV in the US reporting an estimated 75,005 individuals with HDV and a pooled prevalence of 4.2% [15] and a global systematic review reporting anti-HDV prevalence of 4.5% in 120,293 HBsAg positive people [3]. Other studies have found higher HDV prevalence in the US, ranging from 6% in a single-center [16] to 42% in a population-based study of National Health and Nutrition Survey (NHANES) respondents from 2011 to 2016 [9]. The latter study’s sample size is small, consisting of only 113 patients with HBV. However, Gish et al. also report high HBV/HDV infection rates, 38.1%, in Illinois, highlighting that the HDV burden may be much higher than previously appreciated and emphasizing the importance of screening for HDV. Importantly, the NHANES study reports that only 29.5% of patients with HBV/HDV coinfection were even aware that they had liver disease. A global meta-analysis reports an HDV prevalence of 13.02% in HBV carriers and estimates that there are 45-60 million individuals living with HDV worldwide, highlighting the importance of screening given the heightened risk of progressive liver disease in HBV-HDV coinfection [17].

Despite having the fourth highest prevalence of HDV in the US, 5.8% [8], the HDV screening rate in Florida is low at 2.6%. Previously, studies have reported explanations for limited HDV testing in patients with HBV [18,19]. These reasons vary from the lack of a clear definition for an HDV case [18], the lack of universal screening guidelines, discrepancies between providers, and/or the cost of quality testing [16,20]. Our results and the generally low rates of screening for HDV are likely driven by uncertainty about guidelines and knowledge gaps about which tests to order [19]. It is possible that socioeconomic status and access to care influence which patients are diagnosed with HDV. In our study, we found that individuals who were categorized as definite HBV had higher HDV infection rates (14.3%) than those categorized as possible (4.8%) or probable HBV (1.6%). However, all of these participants had chronic HBV and these categories are proxies for access to care. By definition, those with definite HBV had more diagnosis codes and laboratory results, suggesting more engagement with the health system and repeated opportunities to screen for and diagnose HDV. In support of this explanation, a significant number of results from ordered HDV tests were missing in the possible HBV group.

As mentioned above, the AASLD currently recommends screening those at risk for HDV [6], including those with HIV or HCV infection, persons who inject drugs, men who have sex with men, and immigrants from areas of high HDV endemicity [21]. Despite these known risk factors, studies have demonstrated that when the updated EASL HDV testing guidelines were used, there was a significant increase in HDV detection [18,22]. Our study and others [22] reflect a need for updated guidelines to capture the true HDV seroprevalence and reduce HDV transmission.

There are some limitations inherent to analyses of administrative data that could impact generalizability. We utilized strict inclusion criteria and may have excluded participants with chronic HBV without requisite ICD or LOINC codes. Also, OneFlorida+ partners send data at varying intervals. It is possible that some laboratory data were not yet included and could affect the HDV screening or HDV positivity rate. For example, Broward County is one of the most populous counties, yet the number of zip codes mapped to Broward County showed lower than expected prevalence. We intended to identify characteristics associated with HDV infection and determine county-level HDV prevalence but were limited by the small number of completed tests. Despite these limitations, our study of nearly 9,000 individuals with HBV found that HDV seroprevalence in Florida is in line with contemporary estimates of HDV-positivity in the US. Ultimately, this study further highlights the need for improved HDV screening rates to provide a more accurate estimate of HDV disease burden.

## Conclusions

Despite the knowledge that individuals with chronic HBV and HDV have an increased risk of complications, including cirrhosis and hepatocellular carcinoma, screening rates for HDV are low nationally. In Florida, the HDV screening rates are even lower than national averages, suggesting a need for focused attention in this state. The OneFlorida Clinical Research Consortium is a novel resource that permits the localization of patients with chronic hepatitis B and enables a better understanding of practice patterns throughout the state. Despite these low screening rates, HDV seroprevalence is not negligible and approaches 5%, highlighting the need for the implementation of education or awareness campaigns to increase screening and identification of patients with HDV.

Future studies on barriers to HDV screening in Florida, nationally, and globally are needed to improve knowledge of true seroprevalence. Additional studies on how practice environment and local, state, and national geography interact to impact receipt of HDV screening would inform interventions to increase screening. Identifying individuals with HDV could improve care on an individual level and would improve our understanding of their healthcare needs. Ultimately, identifying a larger sample of persons with HDV will enable the development of models informed by Precision Medicine that can refine risk prediction on a population level.

## Appendices

Supplemental Material 1

**Table 3:** International Classification of Diseases (ICD) and Logical Observation Identifiers Names and Codes (LOINC) Codes Used Sources: CMS, International Classification of Diseases, Tenth Revision, Procedure Coding System, ICD-10-PCS, 2024; CMS, Logical Observation Identifier Names and Codes, 2024.

Diagnostic Codes for Chronic Hepatitis B	
Code type	Codes
ICD-09-CM	070.22, 070.23, 070.32, 070.33.
ICD-10-CM	B18.0, B18.1
LOINC Codes for HBV DNA and hepatitis B surface antigen	
LOINC	SHORTNAME
11258-1	HBV DNA Ser-aCnc
20442-0	HBV DNA # Ser Probe+sig amp
23869-1	HBV DNA Ser Probe-mCnc
29610-3	HBV DNA SerPl Ql NAA+probe
29615-2	HBV DNA # SerPl NAA+probe
29900-8	HBV DNA Ser Probe+sig amp-mCnc
32686-8	HBV DNA Ser-mCnc
40459-0	HBV DNA Ser Probe+sig amp-Log#
42595-9	HBV DNA SerPl NAA+probe-aCnc
45161-7	HBV DNA SerPl NAA+probe-Log#
47216-7	HBV DNA # SerPl NAA DL=200
48398-2	HBV DNA SerPl NAA+probe-Log IU
48650-6	HBV DNA # SerPl NAA DL=500
5007-0	HBV DNA Bld Ql NAA+probe
95236-6	HBV DNA SerPl-Imp
95147-5	HBV DNA Pnl SerPl
13126-8	Deprecated HBV DNA Bld Ql IB
59052-1	HIV 1+HCV RNA+HBV DNA SerPl Ql NAA+probe
10674-0	HBV surface Ag Tiss Ql ImStn
10675-7	HBV surface Ag Tiss Ql Orcein Stn
42505-8	HBV surface Ag CSF Ql
47364-5	HBV surface Ag Ser Donr Ql IA
50967-9	HBV surface Ag Bld Donr Ql Nt
51659-1	HBV surface Ag Fld Ql
5195-3	HBV surface Ag Ser Ql
5196-1	HBV surface Ag SerPl Ql IA
5197-9	HBV surface Ag Ser Ql RIA
58452-4	HBV surface Ag Ser-aCnc
63557-3	HBV surface Ag SerPl IA-aCnc
65633-0	HBV surface Ag SerPl Ql Cfm
70154-0	HBV surface Ag SerPl Cfm-%
75410-1	HBV surface Ag SerPlBld Ql IA.rapid
7905-3	HBV surface Ag SerPl Ql Nt
95234-1	HBV surface Ag SerPl-Imp
95149-1	HBV surface Ag Pnl SerPl
LOINC codes for Hepatitis D virus	
LOINC	SHORTNAME
95141-8	Hepatitis D virus Ab panel
95239-0	Hepatitis D virus Ab.IgM panel
95144-2	Hepatitis D virus RNA panel
43281-5	Hepatitis D virus Ag
43280-7	Hepatitis D virus Ag
33464-9	Hepatitis D virus Ag
45159-1	Hepatitis B virus & Hepatitis D virus Ab
22330-5	Hepatitis D virus Ab
13248-0	Hepatitis D virus Ab
9526-5	Hepatitis D virus Ag
72606-7	Hepatitis D virus genotype
5200-1	Hepatitis D virus Ab
5201-9	Hepatitis D virus Ab
95238-2	Hepatitis D virus Ab
40727-0	Hepatitis D virus Ab
51458-8	Hepatitis D virus Ab.IgG
35283-1	Hepatitis D virus Ab.IgG
9525-7	Hepatitis D virus Ab.IgM
34597-5	Hepatitis D virus Ab.IgM
44755-7	Hepatitis D virus Ag
44754-0	Hepatitis D virus Ag
51654-2	Hepatitis D virus Ab
49775-0	Hepatitis D virus Ab.IgG
35273-2	Hepatitis D virus Ab.IgG
44826-6	Hepatitis D virus Ab.IgM
44938-9	Hepatitis D virus Ab.IgM
7906-1	Hepatitis D virus RNA
85512-2	Hepatitis D virus RNA
85513-0	Hepatitis D virus RNA
80426-0	Hepatitis D virus RNA
